# Smartphone applications available to pregnant women in the United Kingdom: An assessment of nutritional information

**DOI:** 10.1111/mcn.12918

**Published:** 2019-12-12

**Authors:** Catherine Bland, Kathryn V. Dalrymple, Sara L. White, Amanda Moore, Lucilla Poston, Angela C. Flynn

**Affiliations:** ^1^ Department of Women and Children's Health, School of Life Course Sciences King's College London London UK

**Keywords:** accuracy, pregnancy, and nutrition, behaviour change techniques, maternal nutrition, smartphone applications

## Abstract

The importance of diet during pregnancy is critically important for the short‐ and long‐term health of both mother and child. The number of apps targeting pregnant women is rapidly increasing, yet the nutritional content of these tools remains largely unexplored. This review aimed to evaluate the coverage and content of nutrition information in smartphone apps available to U.K. pregnant women. Keyword searches were conducted in iTunes and Google Play stores in November 2018. Candidate apps were included if they targeted pregnant women, provided pregnancy‐specific nutritional information, had a user rating of at least 4+ based on at least 20 ratings, and were available in English. Nutritional content was assessed for accuracy against U.K. recommendations. Behaviour change techniques (BCTs) were also evaluated. Twenty‐nine apps were included, seven of which originated in the United Kingdom. There was a large variability in the quality of smartphone app nutritional information. The accuracy of nutrition information varied, and several apps conveyed inappropriate information for pregnancy. On average, 10 BCTs were identified per app (range 2–15). Overall, smartphone apps do not consistently provide accurate and useful nutritional information to pregnant women. This study highlights the need for the integration of evidence‐based nutritional information during app development and for increased regulatory oversight. App developers should also make it clear that nutritional content is intended for a specific geographical region or population or modify for the intended audience. These are important considerations for the design of future apps, which are increasingly used to complement existing maternity services.

**Key Messages**

- This review identified 29 pregnancy‐related apps available to U.K. women and assessed nutritional information in line with national recommendations. There was a large variability in the quality of app nutritional information. The accuracy of nutrition information varied, and several apps conveyed inappropriate information for pregnancy.
- This study highlights the need for the integration of both evidence‐based nutritional information during app development and for increased regulatory oversight to ensure that nutritional content is accurate before it is available for widespread use.
- App developers should also make it clear that nutritional content is intended for a specific geographical region or population or modify for the intended audience. These are important considerations for the design of future apps, which are increasingly used to complement existing maternity services.

## 1 INTRODUCTION

Achieving optimal nutritional intake during pregnancy is essential to maternal and infant health and to the long‐term health of both mother and child (Hanson & Gluckman, 2014). mHealth, defined as the use of mobile and wireless technologies to support achievements of health objectives (World Health Organization, 2011), is widely used to access health and lifestyle information, with the potential to positively impact existing care and reduce dependence on health services (Pal et al., 2013; Whittaker, Mcrobbie, Bullen, Rodgers, & Gu, 2016). Smartphones and mobile applications (apps) have become popular, with more than two thirds of U.K. adults owning a smartphone (Deloitte, 2014). The number of apps targeted at pregnant women is increasing rapidly (Tripp et al., 2014). In an Australian study of 410 pregnant or post‐partum women, three quarters reported using at least one pregnancy app (Lupton & Pedersen, 2016), and in a study of 535 pregnant women in China, almost half of women had used a pregnancy app, with over a quarter reporting that learning about nutrition was the main reason (Wang, Deng, Wen, Ding, & He, 2019). Apps are therefore potentially important digital tools to provide nutritional advice during pregnancy.

Health apps are an underregulated market, and it is important to ensure that nutrition information is evidenced‐based and tailored to the user. To date, the quality of nutrition information included in pregnancy apps has been infrequently explored (Brown, Bucher, Collins, & Rollo, 2018; Womack, Anderson, & Ledford, 2018). Smartphone apps targeted to pregnant women provide an opportunity to promote health behaviours and complement maternity services (Overdijkink et al., 2018). The health care sector must be confident in the alignment of information with evidence‐based recommendations. Behaviour change techniques (BCTs) are defined as observable and replicable components intended to change behaviour (Michie et al., 2015). The incorporation of BCTs indicates the likely efficacy of apps to facilitate behaviour change (Webb, Joseph, Yardley, & Michie, 2010) and has not been widely assessed in pregnancy apps.

The primary aim of this study was to evaluate the quality of nutrition information in popular pregnancy‐related mobile apps to determine the extent to which apps present accurate information for U.K. women. In the United Kingdom, nutritional recommendations during pregnancy are largely in line with population recommendations. There is a modest increment in energy and protein requirements, a need to ensure adequate micronutrient intakes including iron, calcium, iodine, and supplementation of folic acid and vitamin D (Department of Health, 1991; , Scientific Advisory Committee on Nutrition, 2011a, 2011b, 2016, 2017). Because excessive gestational weight gain (GWG) is associated with adverse pregnancy outcomes as well as post‐partum weight retention (Goldstein et al., 2017; Nehring, Schmoll, Beyerlein, Hauner, & Von Kries, 2011), advice on limitation of GWG was also reviewed. A secondary aim of this study was to assess the inclusion of BCTs of included apps.

## 2 METHODS

### 2.1 Search strategy

Keyword searches were conducted in November 2018 on the two leading U.K. platforms: the U.K. iTunes app store (iTunes) and the Google Play Store (GP). To mitigate the influence of device owner and location on search results, searches were made on a personal computer while logged out of any user account and for the GP search, using the private Google Chrome incognito browser. Apps were identified using the following search terms: *nutrition*, *diet*, *food*, *supplement*, *food safety*, *weight gain*, *nutrition recommendations*, *nutrition guidelines*, *vegetarian*, *vegan*, and *gestational diabetes*. Each search term was combined with *pregnancy* and *pregnant* to minimise irrelevant results.

### 2.2 Selection process

Apps were included if they (a) contained any pregnancy‐specific nutritional content and (b) met the eligibility criteria on popularity. To identify the most popular apps, those with an average user rating of ≥4 out of five based on at least 20 ratings were included (Schoeppe et al., 2017). iTunes displays ratings data for both current and all past app versions. Ratings data were considered for all versions as an indicator of longer term app popularity and to avoid bias against newer apps. GP displays number of installs and apps with >500,000 installs have been defined as “popular.” For practical reasons and interpretation of results, apps with <1 million installs were excluded (Franco, Fallaize, Lovegrove, & Hwang, 2016).

Apps were excluded if they (a) only related to the preconception or post‐partum period, (b) had no ratings, (c) were not available in the English language, (d) were forums, or (e) were present in duplicate. This review was not limited to free apps, although app bundles available at a reduced price were excluded and component apps considered individually. Apps with a basic free version and a deluxe version that may contain additional content and functionality that was accessible through “in app” purchases were also considered individually.

### 2.3 Data extraction

Eligible apps were downloaded on either an iPhone or Android device. For each app, name, category, price, country of origin, and date of most recent update were extracted. App content was extracted in three domains: (a) accountability, (b) coverage and accuracy of nutritional content, and (c) incorporation of BCTs.

The apps were assessed for accountability based on Silberg's standards (Silberg, Lundberg, & Musacchio, 1997) and evaluated out of a score of eight, an approach previously used by Chen, Cade, and Allman‐Farinelli (2015). One point was awarded for each of authors credited, authors affiliation, sponsorship disclosure, and if the app had been modified in the last month. Two points were awarded for each of authors credentials and provision of information sources/references.

Coverage and content accuracy of pregnancy‐specific nutrition information in the included apps was evaluated by two authors (C. B. and A. F.). A list of nutrition topics relevant to U.K. pregnant women was classified by expert opinion by three review authors (C. B., A. F., and K. V. D.). Topics included nutrition recommendations and nutrient supplements in line with the U.K. guidelines, food safety recommendations, GWG and pregnancy complications (gestational diabetes [GDM] and pre‐eclampsia [PE]), and information for specific groups (vegetarians, vegans, and adolescents). Weight gain recommendations were assessed using the U.S. National Academy of Medicine (NAM) guidelines (previously known as IOM; Institute of Medicine, 2009). Although these guidelines are not advised for U.K. women, they are widely adopted internationally.

The inclusion of BCTs was assessed using the 26‐item taxonomy of BCTs created by Abraham and Michie (2008). A score of 0 (absent) or 1 (present) was applied for each of the BCTs.

### 2.4 Content analyses

Pregnancy‐specific nutrition content was assessed against the extent that the information agreed or disagreed with national recommendations (Department of Health, 1991; National Institute for Health and Care Excellence, 2010, Scientific Advisory Committee on Nutrition, 2011a, b, 2015, 2016, 2017). This approach has been used for examining the accuracy of online nutrition information for pregnant women (Storr, Maher, & Swanepoel, 2017). Information was categorised as (a) accurate, solely consistent with U.K. recommendations (or NAM guidelines for GWG); (b) mixed, if it was both consistent and inconsistent with U.K. recommendations; and (c) inaccurate, if solely inconsistent with U.K. recommendations.

## 3 RESULTS

### 3.1 App selection

**Figure 1:**
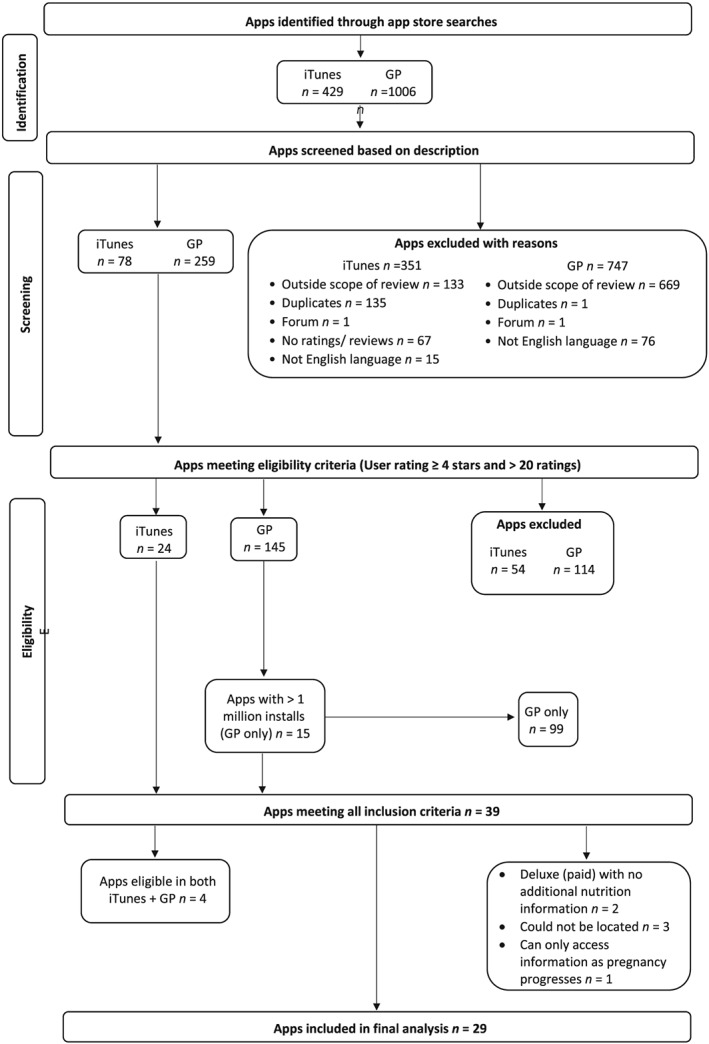
Flowchart of app selection process. GP, Google Play Store

A flowchart of the app selection process is shown in Figure 1. The search strategy identified 1,435 apps (iTunes *n* = 429; GP *n* = 1,006), of which 1,098 (iTunes *n* = 351; GP *n* = 747) were excluded. The remaining 337 apps (iTunes *n* = 78; GP *n* = 259) were further screened for eligibility based on ratings and installs resulting in 39 apps (iTunes *n* = 24; GP *n* = 15). Following further exclusions, a final 29 apps were included in this review.

### 3.2 App characteristics

The apps were predominantly developed in the United States (*n* = 8, 27.6%), followed by the United Kingdom (*n* = 7, 24.1%). Two international apps were “UK” editions (*Sprout Pregnancy* and *Pregnancy Tracker & Countdown to Baby Due Date*). Most of the included apps were categorised within the health and fitness section of the app stores (*n* = 13), followed by medical (*n* = 8), parenting (*n* = 4), shopping (*n* = 1), and education (*n* = 1; Table S1).

### 3.3 Accountability

**Figure 2:**
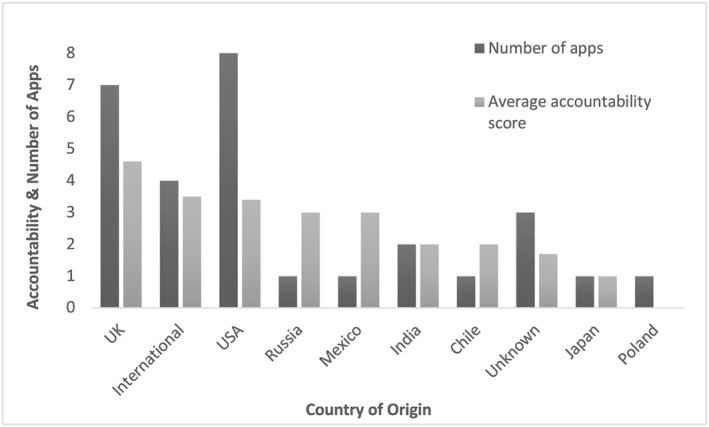
Average accountability score of included apps

The average score for accountability was 3.1 out of eight (range 0–8), with U.K.‐based apps rating the highest (average 4.6) and a Polish app the lowest (0, *n* = 1; Figure 2 and Table S2). Two apps fulfilled all accountability criteria, U.K.‐based *Baby Buddy* and *Emma's diary*. Over half of apps included information sources or references (*n* = 17, 58.6%), whereas fewer than half disclosed sponsorship (*n* = 7, 24.1%).

### 3.4 Nutrition‐specific information

#### 3.4.1 Nutrient and energy recommendations

Information on energy intake in pregnancy was commonly covered among the apps (*n* = 24, 82.8%). The content was assessed against U.K. recommendations (increment of 0.8 MJ day^−1^ [191 kcal day^−1^] in the last trimester only). Out of 15 apps that included energy recommendations in the first trimester, 13 (86.7%) contained accurate information in line with U.K. recommendations, compared with seven of 15 apps (46.7%) in the second trimester and seven of 16 apps (43.8%) in the third trimester. Nine (56.4%) included inaccurate information on energy recommendations in the third trimester (Table 1). Six U.K.‐based apps contained accurate information on energy intake during pregnancy; however, *Mothercare‐for you & baby* app recommended an energy increment in the last trimester of “around 450 kcal for most women.” Information on restricting energy intake in the third trimester was included in *Pregnancy Tracker and Baby Due Date Calculator* (encouraged decreasing caloric intake by 200 calories and fasting 1 day per week).

**Table 1:** App coverage and accuracy of content related to nutrient intakes according to U.K. recommendations

Nutrient	Apps *n* (%)	Recommendation *n* (%)	Accurate *n* (%)	Mixed *n* (%)	Inaccurate *n* (%)
Energy intake	24 (82.8)	20 (83.3)	—	—	—
1st trimester	15 (51.7)	15 (100.0)	13 (86.7)	0 (0.0)	2 (13.3)
2nd trimester	15 (51.7)	15 (100.0)	7 (46.7)	0 (0.0)	8 (53.3)
3rd trimester	16 (55.2)	16 (100.0)	7 (43.8)	0 (0.0)	9 (56.3)
Recommended intakes					
Vitamin A	20 (69.0)	5 (25.0)	1 (20.0)	0 (0.0)	4 (80.0)
Folate	26 (89.7)	7 (26.9)	2 (28.6)	1 (14.3)	4 (57.1)
Vitamin C	18 (62.1)	6 (33.3)	1 (16.7)	1 (16.7)	4 (66.7)
Vitamin D	18 (62.1)	9 (50.0)	3 (33.3)	1 (11.1)	5 (55.6)
Calcium	24 (82.8)	11 (45.8)	2 (18.2)	0 (0.0)	9 (81.8)
Iron	26 (89.7)	10 (38.5)	1 (10.0)	1 (10.0)	8 (80.0)
Iodine	8 (27.6)	4 (50.0)	2 (50.0)	0 (0.0)	2 (50.0)
Supplementation					
Folic acid	26 (89.7)	21 (80.8)	14 (66.7)	4 (19.0)	3 (14.3)
Vitamin D	15 (51.7)	9 (60.0)	7 (77.8)	1 (11.1)	1 (11.1)
Recommended intakes					
Carbohydrate	21 (72.4)	3 (14.3)	1 (33.3)	0 (0.0)	2 (66.7)
Fibre	22 (75.9)	5 (22.7)	0 (0.0)	0 (0.0)	5 (100.0)
Fat	21 (72.4)	4 (19.0)	3 (75.0)	0 (0.0)	1 (25.0)
Protein	24 (82.8)	9 (37.5)	1 (11.1)	0 (0.0)	8 (88.9)

Information on macronutrients was most commonly covered for protein (*n* = 24, 82.8%; Table 1). Out of nine apps that included protein recommendations, only the U.K.‐based *Pregnancy+* specified protein recommendations consistent with the U.K. guidelines of 51 g day^−1^, whereas the remainder of the apps recommended considerably higher intakes ranging from 60 to 100 g per day. Fibre was covered by three quarters of apps (*n* = 22, 75.9%). Five apps included fibre recommendations, but the information was not consistent with the U.K. recommendation of 30 g day^−1^. One U.K.‐based app, *Pregnancy & Birth‐Aptaclub*, gave a fibre recommendation that suggested an intake of 18 g day^−1^ in line with previous, outdated recommendations.

Information on micronutrients was frequently covered for folate (*n* = 26, 89.7%), iron (*n* = 26, 89.7%), and calcium (*n* = 24, 82.8%) and less commonly for iodine (*n* = 8, 27.6%; Table 1). Out of the apps that included nutrient recommendations, the majority included recommendations that were not consistent with the United Kingdom, particularly for calcium (U.K. recommendation 700 mg day^−1^; *n* = 9, 81.8%) and iron (U.K. recommendation 14.8 mg day^−1^; *n* = 8, 80.0%). One app, *Pregnancy Tracker and Baby Due Date Calculator*, recommended an extra intake of 40‐mg iron per day.

Nutrition information on macronutrients and micronutrients was not always appropriate for pregnant women. For example, *Sprout Pregnancy* (encouraged low carbohydrate diets), *Pregnancy Tracker and Baby Due Date Calculator* (“there is a dramatic increase in need for protein […] increase further at 38 weeks”), *Indian Pregnancy & Parenting Tips* (“puffed rice is the best source of carbohydrate” and “eat 13 hard‐boiled eggs to meet vitamin D requirements”), and beef liver was inappropriately recommended as a good source of iron by *Pregnancy Tracker and Baby Due Date Calculator*.

#### 3.4.2 Nutrient supplementation

Nutrient supplementation most commonly described folic acid (*n* = 26, 89.7%), followed by vitamin D (*n* = 15, 51.7%). Out of apps that included information on supplement dose, over two thirds included accurate information on folic acid (U.K. recommendation 400 μg day^−1^ in the first trimester; *n* = 14, 66.7%) and over three quarters included accurate information on vitamin D (U.K. recommendation 10 μg day^−1^; *n* = 7, 77.8%; Table 1). Six U.K.‐based apps included accurate information on both folic acid and vitamin D supplementation dose. Recommended folic acid doses of *at least* 400 mcg (*What to Expect—Pregnancy & Baby Tracker* and *Sprout Pregnancy*) and *at least* 600 mcg (*iPregnant Pregnancy Tracker Free*) were not consistent with U.K. recommendations. Inaccurate information on vitamin D supplementation was included by *Indian Pregnancy & Parenting Tips, The Babycare App* (“only for non‐meat eaters”).

#### 3.4.3 Food and beverage safety issues

Coverage of alcohol consumption during pregnancy was high (*n* = 25, 86.2%), and no apps contained inaccurate information (Table 2). Two apps gave mixed advice. For example, *Bounty pregnancy, birth & baby* advised both that alcohol should be avoided but also included information to limit alcohol consumption to 1–2 units once or twice per week. Information on caffeine intake was also commonly covered (*n* = 22, 75.9%). The majority of apps contained accurate information on limiting caffeine intake to 200 mg day^−1^ (*n* = 16, 72.7%).

**Table 2:** App coverage and accuracy of content related to food safety according to U.K. recommendations

Food/Beverage	Apps *n* (%)	Recommendation *n* (%)	Accurate *n* (%)	Mixed *n* (%)	Inaccurate *n* (%)
Raw or undercook eggs	20 (69.0)	20 (100.0)	18 (90.0)	1 (5.0)	1 (5.0)
Unpasteurised milk and dairy products	20 (69.0)	20 (100.0)	19 (95.0)	0 (0.0)	1 (5.0)
Soft mould‐ripened and blue veined cheeses	14 (48.3)	14 (100.0)	14 (100.0)	0 (0.0)	0 (0.0)
Vit A and containing products	20 (69.0)	17 (85.0)	10 (58.8)	7 (41.2)	0 (0.0)
Raw or undercooked meat	19 (65.5)	19 (100.0)	18 (94.7)	0 (0.0)	1 (5.3)
Undercooked ready prepared dishes	8 (27.6)	8 (100.0)	8 (100.0)	0 (0.0)	0 (0.0)
Unwashed fruit and vegetables	13 (44.8)	13 (100.0)	13 (100.0)	0 (0.0)	0 (0.0)
Caffeine	22 (75.9)	22 (100.0)	16 (72.7)	4 (18.2)	2 (9.1)
Alcohol	25 (86.2)	25 (100.0)	23 (92.0)	2 (8.0)	0 (0.0)
Fish	21 (72.4)	21 (100.0)	13 (61.9)	6 (28.6)	2 (9.5)

The most common food safety topic described fish consumption (*n* = 21, 72.4%), with information to limit oily fish to ≤2 portions per week and fish that may contain high levels of mercury or other pollutants covered by most apps. One app, *Pregnancy Tracker and Baby Due Date Calculator*, contained inaccurate information (advising ≥2 portions of fatty fish per week). Twenty (69.0%) apps included information on foods and supplements containing vitamin A, seven (41.2%) of which included mixed information. *Web MD* recommended liver as a good food source of vitamin A, which is not in line with food safety advice to avoid liver during pregnancy due to its high retinol content. Unpasteurised milk and dairy products were covered by 20 (69.0%) apps, which mostly included accurate information to avoid such products. However, *Happy Pregnancy Ticker app* stated that there is “some concern over listeria, but no evidence to back this up, do some research on risks and benefits.”

#### 3.4.4 Gestational weight gain

The majority of apps contained some information on GWG (*n* = 26, 89.7%; Table 3). Thirteen (81.3%) apps included information on total GWG according to NAM guidelines. Six (46.2%) apps gave accurate information on total GWG, whereas six (46.2%) included inaccurate information. Twelve apps contained information on GWG by trimester, two thirds of which were accurate. Out of the U.K.‐based apps, three apps stated there were no U.K. guidelines, whereas four apps recommended weight gain based on the NAM guidelines. Although weight loss in pregnancy is not advised, *Pregnancy Week By Week* contained inappropriate information that encouraged weight loss (cardio is a “good way to lose weight”).

**Table 3:** App coverage and accuracy of content related to gestational weight gain according to the National Academy of Medicine guidelines

Weight gain	Apps *n* (%)	GWG recommendation *n* (%)	Accurate *n* (%)	Mixed *n* (%)	Inaccurate *n* (%)
Content related to GWG	26 (89.7)	18 (69.2)	9 (50.0)	5 (27.8)	4 (22.2)
Healthy GWG varies by prepregnancy BMI	17 (58.6)	5 (29.4)	3 (60.0)	1 (20.0)	1 (20.0)
Total GWG	16 (55.2)	13 (81.3)	6 (46.2)	1 (7.7)	6 (46.2)
Rate of GWG by trimester	12 (41.4)	12 (100.0)	8 (66.7)	1 (8.3)	3 (25.0)

Abbreviations: BMI, body mass index; GWG, gestational weight gain.

#### 3.4.5 Pregnancy complications

Several apps included nutrition information relating to pregnancy complications including GDM (*n* = 24, 82.8%) and PE (*n* = 21, 72.4%). The information focused mostly on prevention of GDM (*n* = 12, 50.0%) and PE (*n* = 13, 61.9%). Despite there being no national recommendations in relation to nutrition and hypertensive disease during pregnancy, nutritional information on prevention of PE was often provided and highly variable, including avoiding excess weight gain, suggesting that iron supplements increase risk in subjects who are iron sufficient, recommending reducing salt and caffeine intake, and suggesting that risk is increased if deficient in riboflavin, magnesium, and vitamins C, E, and D. The suggestion by two apps (*I'm Expecting—Pregnancy App* and *Pregnancy Tracker & Countdown to Baby Due Date*) that calcium insufficiency may be related to PE is in line with WHO recommendations (World Health Organization, 2018). One app, *Pregnancy Week By Week*, advised “a high protein diet stabilises blood sugar to prevent diabetes,” whereas others recommended to avoid sugar, to consume high fibre meals and fibre supplements, and suggested that iron supplements increase risk in those who are replete (data not shown).

#### 3.4.6 Information for specific groups

A number of apps included nutrition information on vegetarian (*n* = 17, 58.6%) and/or vegan diets (*n* = 14, 48.3%), for which information on protein, iron, calcium, vitamin B12, and vitamin D was commonly given. Iron and vitamin B12 were the most common topics for vegetarian and vegan diets, respectively. Incorrect information on vegan diets was included by *Pregnancy Tracker and Baby Due Date Calculator* (a vegan diet is “too strict, complete rejection of animal protein deprives you and your child of essential amino acids”) and *Pregnancy Week By Week* (“non‐dairy alternatives are just as nutritious,” e.g., almond, coconut, and rice milk). Just five apps (17.2%) contained nutritional information specifically for pregnant adolescents including information on calcium and energy intake (data not shown).

#### 3.4.7 Behaviour change techniques

**Figure 3:**
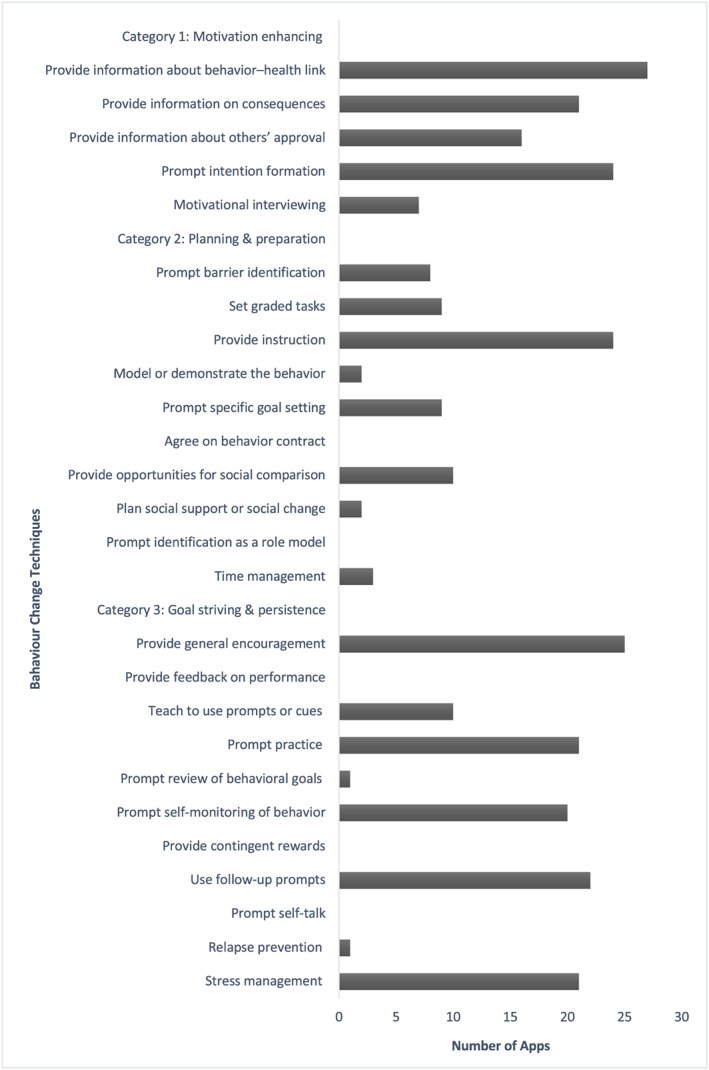
Behaviour change techniques of included apps

All apps included BCTs with an average of 10 techniques out of 26 (range 2–15). *Ovia Pregnancy Tracker: Baby Due Date Countdown* and *Pregnancy+* incorporated the most BCTs, whereas *Ada‐Your Health Guide* and *WomanLog Pregnancy Calendar* had the lowest inclusion. The most common BCT incorporated was *provide information about behaviour‐health link* (*n* = 27, 93.1%), followed by *provide general encouragement* (*n* = 25, 86.2%), *provide instruction* (*n* = 24, 82.8%), and *prompt intention formation* (*n* = 24, 82.8%; Figure 3 and Table S2).

## 4 DISCUSSION

This study demonstrates, for the first time, the large variability in the quality of nutritional information in popular pregnancy‐related apps available in the United Kingdom. The apps varied in accuracy compared with U.K. recommendations, and a number of apps included inappropriate information for pregnant women, some of which could be harmful. This highlights a pressing need for evidence‐driven nutrition guidance for pregnant women via smartphone platforms and a requirement for increased regulation and oversight.

The coverage of nutrition topics relevant to pregnancy varied widely between the apps. Information most commonly described GWG, folic acid supplementation, iron intake, and alcohol consumption. Similarly, Brown et al. recently reported that alcohol was a frequently included topic in a review of pregnancy apps available in Australia (*n* = 32, 62.7%; Brown et al., 2018) and that most common nutrition information described food safety guidelines during pregnancy. Our review also identified a focus on food safety, although a number of apps included mixed information, in line with Womack et al. (2018) who reported conflicting information in prenatal apps. We found that iodine was the least covered nutrition topic, consistent with a review of online nutrition information for Australian pregnant women (Storr et al., 2017). Furthermore, iodine was not widely covered in the context of vegetarian and vegan diets that is of concern due to importance of iodine in fetal brain development (Zimmermann, 2009) and of emerging iodine deficiency in pregnancy (Dahl et al., 2018; Henjum et al., 2018; Snart et al., 2019). Only five apps included pregnancy‐related nutrition information for adolescents, a group who regularly use smartphones (Eurostat, 2017) and are more likely to consume poorer quality diets (Baker et al., 2009). The variation in coverage of nutrition topics may result in women not receiving important information on optimising food and nutrient intakes in pregnancy. The importance of the maternal diet to the short‐ and long‐term health of both mother and child is well recognised (Hanson et al., 2015) and highlights the need for app developers to work with researchers, health professionals, and maternity organisations to provide comprehensive nutrition education for pregnant women, including advice on optimising nutrient intakes with emphasis on key nutrients for pregnancy.

The level of accuracy of nutrition information was a concern, particularly for energy intake during pregnancy, with some apps recommending third trimester energy increments over twice the U.K. recommendation. Inconsistencies in energy and nutrient recommendations were also reported by Storr et al. (2017) where much of the online content was not in line with the Australian guidelines. Some of the inaccuracies may be due to differences in the recommendations in the apps' country of origin compared with the United Kingdom. For example, some apps recommended a calcium intake of 1,000–1,300 mg and an iron intake of 27 mg per day that are in line with the NAM guidelines. The low number of apps providing information on vitamin D supplementation was of concern as the U.K. recommendations clearly state that all women should take a vitamin D supplement during pregnancy (Scientific Advisory Committee on Nutrition, 2016). Although coverage of information on folic supplementation was high, accuracy was lacking in several apps, suggesting that app developers need to work closely with health professionals to ensure that recommendations are modified to be country specific. Apps containing conflicting information may create confusion and anxiety among pregnant women (Wang et al., 2019). The results of the study demonstrate that app developers should make it clear that nutritional content is intended for a specific geographical region or population or modify for the intended audience.

In the absence of U.K. weight gain recommendations, the apps were reviewed for content against the widely used NAM guidelines. Less than half of apps included information on weight gain as defined by the NAM guidelines that is consistent with the findings of Brown et al., where only 16 apps (31%) included the NAM recommendations (Brown et al., 2018). Brown et al. reviewed pregnancy apps available in Australia and suggested that more apps should include the NAM guidelines; however, as they have not been adopted in the United Kingdom (National Institute for Health and Care Excellence, 2010), a number of U.K.‐based apps correctly stated that there were no U.K. recommendations on pregnancy weight gain.

Inclusion of inappropriate information was concerning. For example, although the avoidance of liver is recommended, some apps encouraged its consumption as a good source of vitamins A, B, and iron. Some information on vegan diets was incorrect; the dismissal of vegan diets as “too strict” contradicts the British Dietetic Association who support well‐planned vegan diets during pregnancy. Encouragement of weight loss and fasting during pregnancy by some apps was alarming and has been reported previously (Brown et al., 2018). Furthermore, a number of apps included nutrition information on the prevention of GDM or PE that was not evidenced based. Aside from being potentially harmful, inappropriate information may contradict more reliable sources provided by health care professionals and could create confusion for pregnant women.

The number of BCTs incorporated across the apps varied and was higher than found by a previous review of pregnancy‐related apps (Brown et al., 2018), although a direct comparison is not possible as the 26‐item taxonomy was used in the current study and not the 40‐item taxonomy used by Brown et al. This is encouraging as inclusion of BCTs has been found to be associated with behaviour change (Webb et al., 2010). The most frequent BCTs incorporated were *provide information about behaviour‐health link* and *provide general encouragement* that is similar to a previous review of apps to improve health behaviours of children and adolescents (Schoeppe et al., 2017). No apps incorporated *provide feedback on performance*, which contrasts with weight loss apps (Chen et al., 2015). Evidence suggests that interventions that utilised a combination of self‐monitoring alongside at least one other BCT derived from control theory were more effective compared with other interventions (Michie, Abraham, Whittington, McAteer, & Gupta, 2009). Although *self‐monitoring of behaviour* was incorporated by 21 apps in this review, just five combined this technique with another derived from control theory. App developers should consider using BCTs in the most effective combinations to promote behaviour change.

Accountability varied widely, in particular for apps referencing information sources and disclosing sponsorship. Pregnant women frequently use smartphone apps as a source of health information (Lupton & Pedersen, 2016; Wang et al., 2019). Our findings suggest that the quality of the nutritional content in popular apps available to U.K. women is less than optimal and, in some cases, potentially harmful. It highlights the need for app developers to work closely with health professionals, researchers, and relevant maternity organisations in developing and reviewing evidenced‐based content. Two U.K.‐based apps, *Emma's Diary* and *Baby Buddy*, fulfilled all accountability criteria and contained no inaccurate information. *Baby Buddy* is available in the NHS app library where digital tools are assessed against a range of NHS standards.

The strengths of this study include the comprehensive assessment of pregnancy‐specific nutrition information, drawing upon methods previously used in mHealth reviews. Popularity was taken into account, with apps included only if widely downloaded. Although similar reviews have included only free apps, apps of all prices were included. Finally, the content analysis was completed by two reviewers. Several limitations are acknowledged. Three eligible apps could not be located for download. In‐app purchases were not reviewed. For the GP search, apps with less than one million installs were excluded that could have provided useful data. The 26‐item taxonomy was used to evaluate the inclusion of BCTs, which may have provided a less comprehensive assessment compared with the 40‐item CALO‐RE taxonomy used by a recent review of pregnancy apps in Australia (Brown et al., 2018). The app market is continually evolving, and app versions in this review may have been updated and could be different to currently available versions. Finally, the possibility of assessment bias should be acknowledged.

## 5 CONCLUSION

This review highlights the variability in quality of the nutritional information included in popular apps available to U.K. pregnant women. It is clear from this study that researchers, health professionals, and maternity organisations should work collaboratively with app developers to ensure that nutrition content is comprehensive, accurate, evidence based, and modified for the intended population. This approach would allow women to utilise apps as a reliable nutrition resource in order to make well‐informed dietary decisions to optimise maternal and fetal health. Incorporating evidence from behaviour change interventions could be worthy of further investigation when developing apps. Finally, our work highlights the critical need for more regulation and oversight of mHealth.

## CONFLICT OF INTEREST

The authors declare that they have no conflicts of interest.

## CONTRIBUTIONS

AF designed the study. CB and AF performed the research and analysed the data. All authors interpreted the data. All authors have read and approved the final manuscript.

## Supporting information

Table S1. Characteristics of the included apps

Table S2. Accountability scores and number of BCTs of the included apps
