# Supplementary material for: Smartphone applications available to pregnant women in the United Kingdom: An assessment of nutritional information
Source: Matern Child Nutr. 2019 Dec 12;16(2):e12918. doi: 10.1111/mcn.12918 (PMC7083499; doi:10.1111/mcn.12918)
Supplement: Supplementary file 1 — Table S1. Characteristics of the included apps [file MCN-16-e12918-s001.docx]

**Supplementary Table 1 Characteristics of the included apps**

| **App** | **Price** | **Category** | **Developer** | **Origin/ Developer's location** |
| --- | --- | --- | --- | --- |
| **Pregnancy +** | Free (deluxe £1.99) | Medical | Health & Parenting Ltd | UK |
| **Ovia Pregnancy Tracker: Baby Due Date Countdown** | Free | Medical | Ovuline, Inc. | USA |
| **What to Expect - Pregnancy & Baby Tracker** | Free | Health & Fitness | Everyday Health, Inc. | USA |
| **Sprout Pregnancy** | Free 2 week trial of premium app | Health & Fitness | Med ART Studios | International (UK 'edition') |
| **Pregnancy Tracker & Countdown to Baby Due Date** | Free | Parenting | BabyCenter | International (US owned, UK version) |
| **Pregnancy Week By Week** | Free | Medical | Amila | Unknown |
| **Pregnancy Tracker and Baby Due Date Calculator** | Free (in-app purchases, £2.59) | Medical | Mobile Dimension LLC | Russia |
| **Indian Pregnancy & Parenting Tips,The Babycare App** | Free | Parenting | Healofy | India |
| **I’m Expecting - Pregnancy App** | Free | Health & Fitness | StayWell | USA |
| **280days: Pregnancy Diary** | Free (in-app purchases) | Parenting | Amane factory inc. | Japan |
| **I'm Pregnant - Pregnancy Tracker** | Free | Parenting | BabyJoyApp | Unknown |
| **Happy Pregnancy Ticker** | Free | Health & Fitness | Softcraft | India |
| **Old Pregnancy Gestogram** | Free | Health & Fitness | Isis Producciones y Cia. Ltda | Santiago, Chile |
| **WomanLog Pregnancy Calendar** | Free | Health & Fitness | Pro Active App | USA |
| **Bounty pregnancy, birth & baby** | Free | Health & Fitness | Bounty (UK) Ltd. | UK |
| **Pregnancy & Birth - Aptaclub** | Free | Health & Fitness | Danone Holdings UK | UK |
| **Glow Nurture - Pregnancy App** | Free (in-app purchases £7.99) | Health & Fitness | Glow, Inc. | USA |
| **Pregnancy Workouts - Baby2Body** | Free (in-app purchases £6.49) | Health & Fitness | Baby2Body Limited | UK |
| **iPregnant Pregnancy Tracker Free (iPeriod's Pregnancy Companion)** | Free (deluxe £3.99) | Health & Fitness | Winkpass Creations, Inc. | USA |
| **WebMD Pregnancy** | Free | Health & Fitness | WebMD | USA |
| **Baby Buddy - Pregnancy Guide** | Free | Medical | Best Beginnings | UK |
| **Pregnancy and Due Date Tracker** | Free | Medical | Wachanga Inc | International (USA/ Russia/ Ukraine) |
| **HiMommy - Pregnancy Tracker** | Free | Lifestyle | Idea Accelerator | Poland |
| **Pregnancy Today - Baby Tracker** | Free (in-app purchases) | Health & Fitness | Mushroom Apps | Unknown |
| **Mom.life - pregnancy and baby** | Free | Medical | Wunderkind Media and Technology Corporation | USA |
| **Mothercare - for you & baby** | Free | Shopping | Mothercare UK Ltd. | UK |
| **Emma's Diary** | Free | Lifestyle | Lifecycle Marketing (Mother & Baby) Ltd | UK |
| **Ada - Your Health Guide** | Free | Medical | Ada Health GmbH | International (Based in Berlin, Offices in Germany, USA, UK) |
| **Kinedu: Baby Development App** | Free (in-app purchases available) | Education | Kinedu SAPI de CV | Mexico |
