# Supplementary material for: Smartphone applications available to pregnant women in the United Kingdom: An assessment of nutritional information
Source: Matern Child Nutr. 2019 Dec 12;16(2):e12918. doi: 10.1111/mcn.12918 (PMC7083499; doi:10.1111/mcn.12918)
Supplement: Supplementary file 2 — Table S2. Accountability scores and number of BCTs of the included apps [file MCN-16-e12918-s002.docx]

**Supplementary Table 2 Accountability scores and number of BCTs of the included apps**

| **App** | **Accountability score (out of 8)** | **Number of BCTs** |
| --- | --- | --- |
| **Pregnancy +** | 2 | 12 |
| **Ovia Pregnancy Tracker: Baby Due Date Countdown** | 5 | 16 |
| **What to Expect - Pregnancy & Baby Tracker** | 3 | 14 |
| **Sprout Pregnancy** | 5 | 13 |
| **Pregnancy Tracker & Countdown to Baby Due Date** | 5 | 12 |
| **Pregnancy Week By Week** | 1 | 10 |
| **Pregnancy Tracker and Baby Due Date Calculator** | 3 | 11 |
| **Indian Pregnancy & Parenting Tips,The Babycare App** | 2 | 7 |
| **I’m Expecting - Pregnancy App** | 4 | 11 |
| **280days: Pregnancy Diary** | 1 | 7 |
| **I'm Pregnant - Pregnancy Tracker** | 3 | 9 |
| **Happy Pregnancy Ticker** | 2 | 11 |
| **Old Pregnancy Gestogram** | 2 | 10 |
| **WomanLog Pregnancy Calendar** | 2 | 2 |
| **Bounty pregnancy, birth & baby** | 5 | 10 |
| **Pregnancy & Birth - Aptaclub** | 4 | 9 |
| **Glow Nurture - Pregnancy App** | 6 | 12 |
| **Pregnancy Workouts - Baby2Body** | 5 | 11 |
| **iPregnant Pregnancy Tracker Free (iPeriod's Pregnancy Companion)** | 1 | 8 |
| **WebMD Pregnancy** | 5 | 12 |
| **Baby Buddy - Pregnancy Guide** | 8 | 15 |
| **Pregnancy and Due Date Tracker** | 2 | 8 |
| **HiMommy - Pregnancy Tracker** | 0 | 8 |
| **Pregnancy Today - Baby Tracker** | 1 | 7 |
| **Mom.life - pregnancy and baby** | 1 | 8 |
| **Mothercare - for you & baby** | 0 | 7 |
| **Emma's Diary** | 8 | 9 |
| **Ada - Your Health Guide** | 2 | 2 |
| **Kinedu: Baby Development App** | 3 | 12 |
